# Electrical Changes in Polish Patients with Chronic Heart Failure: Preliminary Observations

**DOI:** 10.3390/medicina55080484

**Published:** 2019-08-15

**Authors:** Grzegorz Sobieszek, Radosław Mlak, Aneta Skwarek-Dziekanowska, Aneta Jurzak-Myśliwy, Iwona Homa-Mlak, Teresa Małecka-Massalska

**Affiliations:** 1 Clinic of Cardiology and Internal Medicine, Department of Cardiology, Military Hospital, 20-093 Lublin, Poland; 2 Human Physiology Department, Medical University of Lublin, 20-080 Lublin, Poland

**Keywords:** chronic heart failure (CHF), bioelectrical impedance analysis (BIA), electrical changes, phase angle (PA)

## Abstract

*Introduction*: Direct parameters resistance (R), reactance (Xc), phase angle (PA), capacitance of membrane (Cm), and impedance ratio (Z200/Z5)) determined by bioelectrical impedance analysis (BIA) detect changes in tissue electrical properties and have been found to be a marker of cell membrane function in various diseases. *Materials and Method*: The cross-sectional study was conducted to investigate whether direct bioimpedance parameters differ in a group of heart failure (HF) patients divided on the basis of the New York Heart Association (NYHA) functional classes I–II and III–IV. BIA was evaluated in 100 patients with HF treated in Clinic of Cardiology and Internal Medicine, Department of Cardiology, Military Hospital, Lublin. *Results*: In men, lower PA values (*p* = 0.01), Xc (*p* < 0.01), Cm (*p* = 0.02), and higher values of the Z200/Z5 ratio (*p* < 0.01) were observed in patients classified into NYHA groups III and IV in comparison to those with lower stages of disease. Similar correlations were noted in women (only Cm differences were insignificant). In addition, in men, C-Reactive Protein (CRP) correlated negatively with PA (*p* < 0.01), Xc (*p* < 0.01), and Cm (*p* < 0.01) and positively with the Z200/Z5 index (*p* < 0.01). There were no similar correlations observed in women. *Conclusion*: Patients with advanced CHF have altered electrical values. Changes in electrical values may directly reflect tissues as well as the whole-body condition.

## 1. Introduction

The electrical properties of altered cells (like cancer cells or cells changed by the inflammatory process) are different than in the normal tissues. Cells of the body possess electrical mechanisms and use electricity to regulate and control the transduction of chemical energy and other life processes. The electrical changes may precede biochemical disorders and, thus, also clinical symptoms of many diseases [1,2].

Chronic heart failure (CHF) involved 1% to 2% of the adult population in developed countries. This percentage increases in the group above 70 years old and is estimated to be over 10% [3].

The most common symptoms of CHF are associated with fluid retention and are the following: dyspnea, orthopnea, decreased exercise tolerance, and gravitational edema. Less characteristic symptoms such as tachycardia, palpitations, weight gain, dizziness, and depression are difficult to assess especially in elderly patients with chronic lung diseases and other chronic diseases [3].

The severity of symptoms helps doctors to make decisions about further therapy, especially invasive treatment. Many factors related with poor prognosis have been taken into consideration in patients with CHF. However, the risk assessment with their use remains very limited. Studies evaluating many different prognostic models showed only their moderate usefulness in the assessment of prognosis in patients with CHF [4,5]. CHF is well known to be an heterogenous syndrome in which an inflammatory process plays a great role, especially in the disease progression, severity, and prognosis. Inflammatory cytokines participate in CHF pathophysiology in many ways, which leads to hyperthrophy, fibrosis, and impaired cardiac contractility [6].

Bioelectrical impedance analysis (BIA) evaluates body properties e.g., impedance (Z), reactance (Xc), and resistance (R) at different frequencies (5, 10, 50, and 500 kHz) by recording voltage change in the applied current [7].

Another phase angle (PA) is determined on the basis of the R and Xc values obtained by BIA, according to the formula PA = arc tangent Xc/R [8]. The PA is considered to be an indicator of the cell condition and it closely correlates with the cellular mass of the body (BCM) [9]. The value of the PA depends on the integrity of cell membranes and the electric potential difference on both sides. As the higher value of the PA, than the cells’ function is better. Furthermore, the value of this parameter reflects the efficiency of energy processes and proteolysis, which indicates the patient’s well-being. The studies carried out so far have allowed us to determine population norms for the PA. For healthy adults, it is 5–7°, and the value below 5° may indicate malnutrition [9]. However, all clinical data should be taken into consideration because many diseases may alter the PA value. The PA is a diagnostic and prognostic parameter in many chronic diseases [10,11,12,13,14,15,16]. The standard frequencies used for BIVA (Bioelectrical impedance vector analysis) include 5, 50, 100, and 200 kHz. The additional parameter is the whole-body impedance ratio at 200 kHz to 5 kHz (Z200/Z5), which is served as an indicator of water distribution. In the current studies, close correlation of the ratio Z200/Z5 with the higher functional class of New York Heart Association (NYHA) symptoms has been demonstrated [17].

A further parameter, which is derived from BIA, is capacitance of the membrane (Cm). The Cm is considered to be a physical quantity equal to the ratio of charge collected on the conductor to the potential of the conductor [18]. BIA is a well-established tool of objective evaluation of body composition and, thus, nutritional status in different diseases such as cancer [19,20,21,22]. The utility of these tools has been assessed by their ability to predict different clinical outcomes such as: treatment response, complications, quality of life (QoL), and survival [22,23]. Many BIA parameters were compared between each other to evaluate the ability of prediction of different clinical outcomes, but only few evaluate the ability to predict overall survival [4].

In all chronic inflammation diseases, the parameter that is assessed is C-Reactive Protein (CRP). The level of CRP correlates with many chronic and acute diseases. In patients with acute and CHF, CRP concentrations changed without infection and its concentrations may be a prognostic marker [24].

In the Polish population, there are no studies evaluating electrical parameters derived from BIA in patients with CHF. This prospective study was conducted to investigate the difference in selected electrical parameters in those patients with correlation to the inflammatory status.

## 2. Materials and Methods

All procedures performed in studies involving human participants were in accordance with the ethical standards of the institutional and/or national research committee and with the 1964 Helsinki declaration and its later amendments or comparable ethical standards. The study project was accepted by the Research Ethics Committee of the Medical University of Lublin, Poland (consent no.: KE-0254/64/2017). Written informed consent to participate in the study was obtained from all patients. This article does not contain any studies with animals performed by any of the authors. Informed consent was obtained from all individual participants included in the study.

The study group consists of 100 patients with CHF divided on the basis of New York Heart Association (NYHA) functional classes I–II and III–IV. All patients were treated at the Clinic of Cardiology and Internal Medicine, Department of Cardiology, Military Hospital, Lublin, Poland between January 2017 and January 2018. Study enrollment criteria: (a) at least 18 years old, (b) obtain of informed consent before study entry, (c) lack of metallic implants, (d) the presence of all limbs, and (e) the absence of the cardioverter or defibrillator.

### 2.1. Outcome Measures

In all patients, data evaluation, including: demographic (sex, age), clinical (NYHA stage), laboratory (CRP), and BIA measurements (PA, Xc, R, Cm, Z200/Z5) was performed. Every time prior to consultation, a physician reviewed the patient’s medical record and verified any change in patient’s weight. BIA was conducted using ImpediMed bioimpedance analysis SFB7 BioImp v1.55 (PinkenbaQld 4008, Australia). During BIA, patients were lying supine and their legs and arms were not touching the torso. All measurements were performed on the patients’ right side. The four-surface standard tetra polar electrodes technique on the foot and hand was used. R and Xc were measured three times in each patient (mean values were than calculated), directly in Ω at 50 kHz. Cm and PA, Z5, and Z200 values were automatically obtained from the equipment. The Z200/Z5 ratio was calculated.

### 2.2. Statistical Analysis

The statistical calculations were performed using Statistica 13 PL (StatSoft, Palo Alto, CA, USA) and MedCalc 15.8 (MedCalc Software, Ostend, Belgium) computer software. The difference was considered to be statistically significant if *p* ≤ 0.05. Continuous variables such as PA50, Xc50, R50, Z200/Z5, Cm, and CRP were compared according to NYHA functional classes I–II and III–IV of CHF using the non-parametric U test-Manna-Whitney test. Correlation of this type of variable was assessed using the Spearman rank correlation test.

## 3. Results

In the study group, there was 44 women and 56 men. Median age of patients was 73 years. Both in men and in women, most patients had 3rd degree (according to NYHA scale) of CHF (30.4% and 36.4%, respectively). Mean value of Body Mass Index (BMI) and weight indicate that most patients were overweight (BMI: 29.2 ± 5.9, weight: 81.9 ± 17.1 kg). Baseline characteristics of the study group was presented in Table 1.

**Figure 1:**
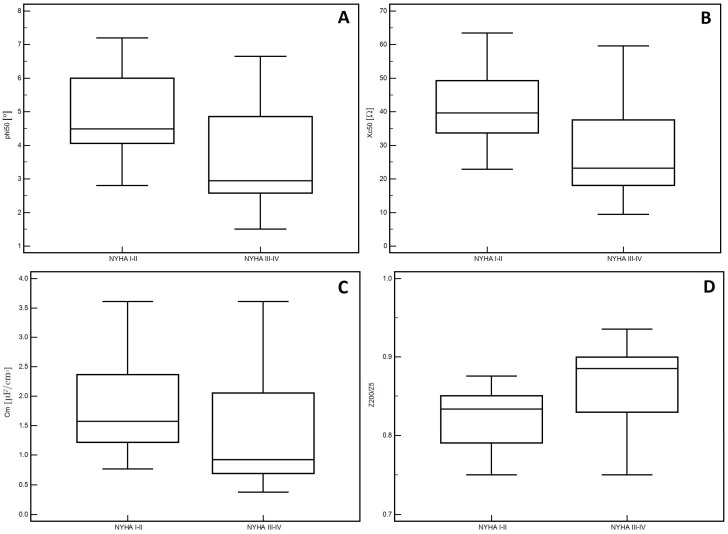
Differences in the values of electrical parameters obtained from BIA depending on the severity of chronic heart failure according to NYHA scale in men. (**A**) Differences between phase angle at 50 kHz (PA), (**B**) reactance at 50 kHz (Xc), (**C**) capacitance of membrane (Cm) and (**D**) impedance ratio (Z200/Z5) depending on the severity of chronic heart failure according to New York Heart Association (NYHA) scale in men.

**Figure 2:**
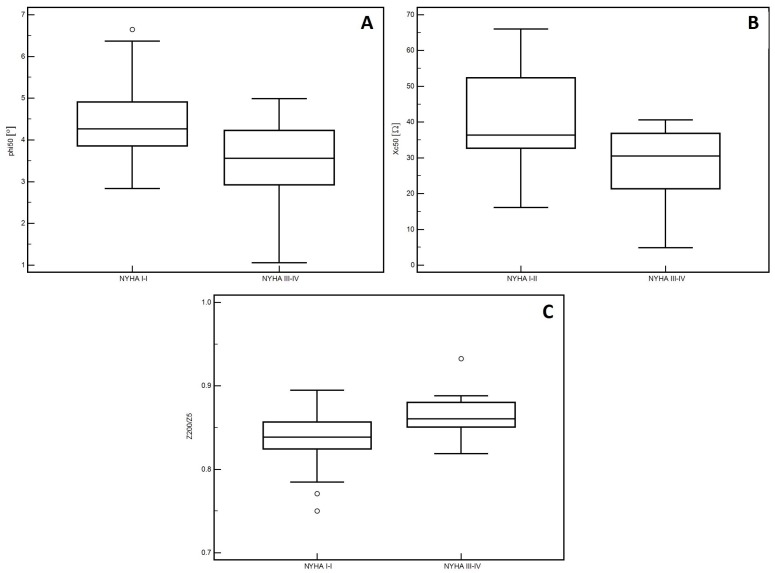
Differences in the values of electrical parameters obtained from BIA depending on the severity of chronic heart failure according to the NYHA scale in women. (**A**) Differences between phase angle at 50 kHz (PA). (**B**) reactance at 50 kHz (Xc). (**C**) impedance ratio (Z200/Z5). depending on the severity of chronic heart failure according to the New York Heart Association (NYHA) scale in women.

**Figure 3:**
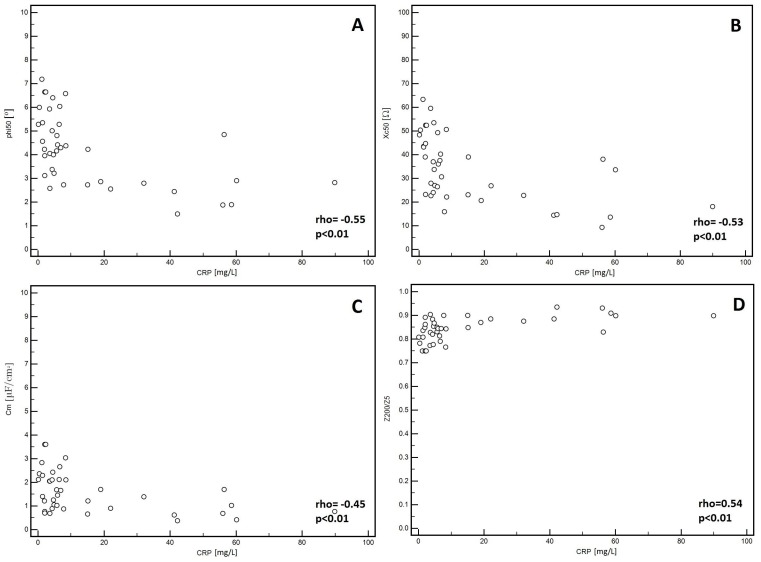
Correlation between CRP and electrical parameters obtained from BIA in men. (**A**) Correlation between C-reactive protein (CRP) and phase angle at 50 kHz (PA) in men. (**B**) reactance at 50 kHz (Xc) in men. (**C**) capacitance of membrane (Cm) in men. (**D**) impedance ratio (Z200/Z5) in men.

**Figure 4:**
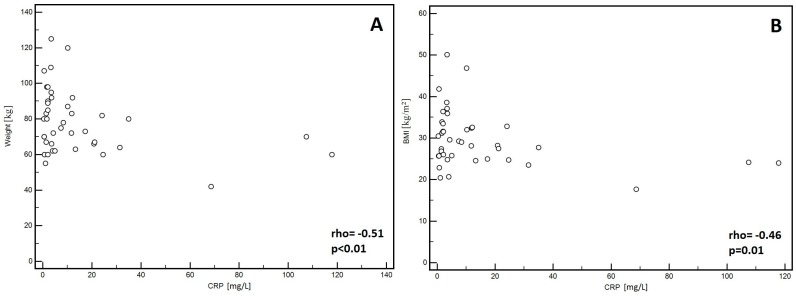
Correlation between C-reactive protein (CRP) and body mass index (BMI) (**A**) as well as with body weight (**B**) in women.

In the men, significantly lower PA values (*p* = 0.01, Figure 1A) and Xc (*p* < 0.01, Figure 1B) were observed in patients classified into NYHA groups III and IV compared to those with lower stages of disease (I and II). Similarly, lower Cm values were noted in patients with higher stages of disease (III and IV: *p* = 0.02, Figure 1C). However, significantly higher values of the Z200/Z5 index were observed in patients with higher stages of disease (III and IV: *p* < 0.01, Figure 1D). Detailed data on the comparison between electrical measurements obtained on the basis of BIA depending on the severity of chronic HF in men was presented in Table 2. Similarly, in the women, significantly lower PA values (*p* = 0.02, Figure 2A) and Xc (*p* = 0.03, Figure 2B) were observed in patients classified into NYHA groups III and IV as compared to those with lower stages of disease (I and II). However, significantly higher values of the Z200/Z5 ratio were observed in patients with higher stages of disease (III and IV: *p* = 0.02, Figure 2C). Detailed data on the comparison between electrical measurements obtained on the basis of BIA depending on the severity of CHF in women was presented in Table 3. Moreover, in men, CRP correlated significantly with PA (*p* < 0.01, Figure 3A), Xc (*p* < 0.01, Figure 3B), and Cm (*p* < 0.01, Figure 3C). A positive correlation with this factor was observed only in the case of Z200/Z5 ratio (*p* < 0.01, Figure 3D). In women, CRP significantly correlated negatively only with BMI (*p* = 0.01, Figure 4A) and body weight (*p* < 0.01, Figure 4B). Detailed data on the correlation between CRP and other studied factors was presented in Table 4.

## 4. Discussion

In recent decades, heart failure (HF) is still a growing problem. HF is a chronic, progressive process and is often the consequence of most heart diseases. It is characterized by high morbidity, mortality, and frequent exacerbations requiring hospitalization [17]. This is paradoxically due to the greater possibilities of pharmacological and surgical treatment of ischemic heart disease, more effective treatment of valvulopathy, and prolonged survival of patients [25]. The incidence of HF increases rapidly with age. It is a health problem that affects not only patients but also their families and the health care system. The highest costs are generated by hospitalizations related to exacerbations and rehospitalizations, which concern almost half of patients with HF [26]. For this reason, new methods should be sought to predict the risk of exacerbation in patients with CHF in a non-invasive, objective, and easily understandable manner. Symptoms of HF are mainly caused by fluid retention in the body. It is caused by renin-angiotensin-aldosterone (RAA) activation, which aims to restore homeostasis in the body. The negative effect of RAA activation is poor remodeling of the heart, water retention, and electrolyte disturbances [27]. There is increasing evidence that body composition should be considered as a systemic marker of disease severity in CHF [17,28]. Moreover, there is a lot of evidence in the current literature that pro-inflammatory cytokines and CRP are a prognostic factor in CHF [29,30].

For a long time, it has been known that the electrical properties of patients with CHF cells are different than the electrical properties of the healthy man [9,17]. Based on this information, we postulate new cell condition markers among patients with CHF. BIA can be the method of choice in diagnostic of CHF because it is easy, repeatable, and non-invasive. Increased fluid retention changes the electrical properties of cells, which can be monitored with BIA [9,17]. BIA evaluates body properties such as resistance (R) and reactance (Xc) by recording a voltage drop in applied current. BIA has also been recognized as an important tool in the objective evaluation of body composition and nutritional status in many diseases including cancer. BIA parameters differ by many factors. From measured R and Xc values, various body compartments can be assessed by use of specific prediction equations that are based on model assumptions, and that, in general, are population-specific, age-specific, sex-specific, fatness-specific, and disease-specific [11].

The first parameter of BIA that we assessed was the PA. As we previously mentioned, the PA is a parameter indicating the health of cells and its higher value indicates better cell functioning. It was found that PA is a prognostic marker in several clinical conditions, such as the cirrhosis, human immunodeficiency virus infection, chronic obstructive pulmonary disease (COPD), hemodialysis, sepsis, and cancer [10,11,12,13,14,15,16]. The PA as an independent prognostic factor in CHF has not been determined but the lower value is associated with a worse NYHA class, more frequent anemia, and thyroid disease in these patients [31,32]. In our study, we observed a decrease in the value of the PA with the higher stage in the NYHA classification.

In comparison, in 2011, Colin-Ramirez et al., retrospectively, examined 389 patients with HF and the end point was death for any reason. Due to the PA values, patients were classified according to quartiles into four groups ((1) PA < 4.2°, (2) PA 4.2–4.9°, (3) PA 5.0–5.6°, and (4) PA > 5.7°). They found that patients below the lowest quartile of PA (<4.2°) had decreased mean BMI, handgrip strength, and hemoglobin values and a larger proportion of patients in NYHA functional class III and renal failure. Colin-Ramirez et al. showed better survival of patients in the group of the highest quartiles of PA (>5.7°) and shorter survival as the PA decreases. The value of the PA <4.2° was an independent predictor of mortality (relative risk 3.08, 95% CI: 1.06–8.99) in comparison with PA 5.7° [33].

In another study, Martinez et al. studied 243 patients with HF (140 with systolic dysfunction (HFS) and 103 with preserved systolic function (HFPSF)) and comparing them according to the functional NYHA class. In both groups, HFS and HFPSF, the PA was significantly lower in patients in NYHA III-IV than NYHA I-II (4.8 vs. 5.8° in men and 4.2 vs. 4.9° in women) [17].

The significance of the PA was also studied in COPD. In 2018, Blasio et al. in a prospective study examined a group of 263 patients (185 men, 78 women). They discovered that patients with diagnosed malnutrition had significantly lower PA values as compared to those with a normal nutritional status (median: 4.69 and 4.87°). The PA values were the lowest in malnourished patients with advanced severe inflammation (PA = 4.11°) (*p* < 0.05). Similar observations were noted in patients with current sarcopenia. Median PA for patients without sarcopenia 4.90 and 4.66° with sarcopenia and 4.29° with severe sarcopenia (*p* < 0.05) [34].

The importance of the PA was also studied in liver disease. In 2018, Bering et al. examined 135 patients (68 men and 67 women) with hepatitis C. They studied the nutritional status of patients using the Subjective Global Assessment (SGA) scale for the risk of developing cirrhosis. A low PA value was associated with high risk of developing liver cirrhosis (OR = 3.92, *p* < 0.01) and high risk of malnutrition (OR = 5.52, *p* = 0.05) [35].

According to the study, Olivier et al. carried out a prospective study on a group of 58 hemodialyzed patients (28 men and 30 women). They discovered that the PA negatively correlates with the SGA score and positively with the albumin level, percent of standard body weight, arm circumference, and body fat mass (BFM) [36]. Moreover, in 2003, Mushnick et al. examined 48 patients with peritoneal dialysis. They found that patients with PA greater than 6 degrees had a better prognosis of two years’ survival [37].

The studies we mentioned above suggest PA to be a valuable marker and prognostic factor in various diseases changing electrical and functional properties, including HF.

Similarly, changes in PA value and lower Xc values were observed in patients classified into NYHA groups III and IV (*p* < 0.01) compared with lower stages of disease. Similar conclusions in our study were achieved in 2007 by Martinez et al. They observed significantly lower values of Xc in NYHA III-IV group regardless of sex and age [17].

An additional parameter that can be determined using bioimpedance is Cm. The Cm is a physical quantity equal to the ratio of charge collected on the conductor to the potential of the conductor. The Cm describes the measure of the oscillating current that is caused by the flow of electric ions across the cell membrane [19]. So far, no work has been created to determine diagnostic and prognostic values of this parameter in CHF. In our study, lower Cm values were observed in patients in the NYHA functional class III-IV and observed to increase in NYHA functional class I-II (*p* = 0.02). However, the differences were observed only in men. Although it needs further studies, Cm can be regarded as another remarkable CHF marker despite PA.

Małecka-Massalska et al. examined a cohort of 75 stage IIIB and IV head and neck cancer (HNC) patients. This prospective study assessed the effect of Cm value on survival patients suffering from malnutrition and the well-nourished (According to Subjective Global Assessment Scale, SGA) adult patients with HNC. A significantly higher median Cm was observed in well-fed patients (*n* = 45) compared to poorly nourished (*n* = 30) (1.41 vs. 1.01 1 µF/cm^2^, *p* < 0.01). A lower Cm below the level of 0.743 was associated with a significantly higher risk of OS shortening than in other patients (12.1 and 43.4 months respectively. HR = 8.47, 95% CI: 2.91–24.66; χ^2^ = 15.38, *p* < 0.01) [8].

The next parameter we specified was Z200/Z5. This is a parameter of water distribution and values closer to 1.00, which are indicative of a significantly deteriorating cellular function. In our study, the impedance ratio Z200/Z5 is higher in the NYHA III-IV. Similar results have been achieved by Martinez et al. in the study we mentioned above. They found that the NYHA III-IV group had a greater ratio Z200/Z5 both in men (group of 51 patients with HFPSF and 82 patients with HFS) and women (group of 52 patients with HFPSF and 58 patients with HFS) (women 0.85 NYHA III-IV versus 0.82 NYHA I-II, men 0.83 versus 0.80, *p* < 0.01) [17]. Itobi et al. also studied the impedance ratio Z200/Z5. They observed 38 patients (21 men and 17 women) for the occurrence of edema after major abdominal surgery and reported that the ratio Z200/Z5 was higher in those patients who develop edema than in patients without (0.81/0.03 in group with edema versus 0.80/0.02 in group without, *p* = 0.01) [36]. The Z200/Z5 can be especially an important marker of fluid retention and an early marker of HF exacerbation and allow for an appropriate intensification of pharmacological treatment and avoid future hospitalization.

In addition to our study, the results of BIA were referred to CRP. As we have already mentioned, CRP is a prognostic factor in HF, which plenty of evidence in the available literature supports [29,30]. Our results show that in men, CRP negatively correlates with PA (*p* < 0.01), Xc (*p* < 0.01), and Cm (*p* < 0.01) and positively correlates with the Z200/Z5 ratio (*p* < 0.01). In women, CRP correlates negatively with BMI and body weight (*p* < 0.01). In our study, CRP correlated only with BIA parameters in men. The reason might be the number of patients, which was the study limitation. Usually, CRP correlates with the inflammation state, which is also the background of malnutrition, especially when chronic. We expected to observe the correlation of CRP and BIA parameters in both groups (men and women).

This is one of the first studies addressing this problem in the Polish population. Initially, we examined only a small population of patients (100 patients) with CHF hospitalized in a single specialized department. Further research is necessary to determine cut-off points of the main parameters and define groups of patients at the risk of exacerbation. It seems that, due to the repetitive and non-invasive method, that BIA will be able to assess the risk and predict an exacerbation in patients with CHF, which will reduce the number of hospitalizations and the cost of treatment of patients with CHF.

## 5. Conclusions

Patients with advanced CHF have altered electrical values. Regardless of the gender, patients with advanced CHF have lower PA and Xc values and higher Z200/Z5 ratios. Cm values are lower in advanced disease only in men. In men, CRP negatively correlates with PA, Xc, and Cm. Moreover, CRP positively correlates with the Z200/Z5 ratio. In women, CRP correlates negatively with BMI and body weight. Therefore, observed in patients, changes in electrical values may directly reflect tissues as well as the whole-body condition. BIA parameters can be, therefore, regarded as CHF deterioration markers although it needs further studies.

## Figures and Tables

**Table 1:** Baseline characteristic of patients with chronic heart failure.

Factor	Study Group (*n* = 100)		*p*-Value
Gender	Male	Female	
	56 (56%)	44 (44%)	
Age (years)	70.5 (43–109) ± 13.91	77 (27–95) ± 11.24	0.08
*Me* (min–max) ± SD			
NYHA stage			
I	13 (23.2%)	9 (20.5%)	0.45
II	15 (26.8%)	15 (34.1%)	
III	17 (30.4%)	16 (36.4%)	
IV	11 (19.6%)	4 (9.1%)	
Weight (kg) Mean ± SD	84.60 ± 16.51	78.25 ± 17.19	0.07
BMI Mean ± SD	28.61 ± 5.34	29.81 ± 6.60	0.34
CRP (mg/L) Mean ± SD	16.06 ± 22.34	14.72 ± 25.93	0.61

*Me*, Median, SD, standard deviation; NYHA, New York Heart Association; BMI, body mass index; CRP, C-reactive protein.

**Table 2:** Differences in electrical parameters obtained on the basis of Bioelectrical Impedance Analysis (BIA) depending on the severity of chronic heart failure according to the New York Heart Association (NYHA) scale in men.

Variable	NYHA I–II *Me* (min–max)	NYHA III–IV *Me* (min–max)	*p*-Value
phi50 (^o^ at 50 kHz)	4.49 (2.80–7.19)	2.95 (1.50–6.65)	0.01
Xc50 (Ω at 50 kHz)	39. (22.87–63.44)	23.17 (9.39–59.55)	<0.01
R50 (Ω at 50 kHz)	480.31 (363.22–480.31)	419.43 (286.75–662.30)	0.09
Z200/Z5 (ratio at 50 and 200 kHz)	0.83 (0.75–0.88)	0.89 (0.75–0.94)	<0.01
Cm (1 µF/cm^2^)	1.57 (0.77–3.61)	0.92 (0.38–3.61)	0.02

*Me*—Median; phi50, Phase angle at 50 kHz; Xc50, Reactance at 50 kH; R50, resistance at 50 kHz; Z200/Z5, Impedance ratio (at 200 and 5 kHz); Cm, capacitance of membrane.

**Table 3:** Differences in electrical parameters obtained on the basis of bioelectrical impedance analysis (BIA) depending on the severity of chronic heart failure according to the New York Heart Association (NYHA) scale in women.

Variable	NYHA I–II (0) *Me* (min–max)	NYHA III–IV (1) *Me* (min–max)	*p*-Value
phi50 (^o^ at 50 kHz)	4.27 (2.83–6.65)	3.56 (1.06–4.98)	0.02
Xc50 (Ω at 50 kHz)	36.37 (16.08–65.97)	30.54 (4.83–40.61)	0.03
R50 (Ω at 50 kHz)	472.02 (309.11–673.90)	457.4273 (261.64–606.39)	0.17
Z200/Z5 (ratio at 50 and 200 kHz)	0.89 (0.75–0.89)	0.86 (0.82–0.93)	0.02
Cm (1 µF/cm^2^)	1.42 (0.54–3.61)	1.14 (0.71–1.98)	0.16

*Me*—Median.

**Table 4:** Correlation between C-reactive protein (CRP) and electrical and body composition parameters.

Variable	CRP	
	Men	Women
	Rho	Rho
	*p*-Value	*p*-Value
Phase angle (ph) 50 (^o^)	−0.55	−0.13
	*p* < 0.01	*p* = 0.51
Resistance (R) 50 (Ω at 50 kHz)	−0.14	−0.0004
	*p* = 0.41	*p* = 1.00
Reactance (Xc) 50 (Ω at 50 kHz)	−0.53	−0.10
	*p* < 0.01	*p* = 0.60
Capacitance of membrane (Cm) (1 µF/cm^2^)	−0.45	0.29
	*p* < 0.01	*p* = 0.14
Impedance ratio Z 200/Z5 (at 50 and 200 kHz)	0.54	0.02
	*p* < 0.01	*p* = 0.91
Total body water (TBW) (in liters)	−0.0629	−0.24
	*p* = 0.70	*p* = 0.23
Total body water (TBW%)	0.26	0.48
	*p* = 0.11	*p* = 0.01
Extracellular fluid (ECF) (in liters)	0.2	−0.20
	*p* = 0.23	*p* = 0.31
Extracellular fluid (ECF%)	0.53	0.04
	*p* < 0.01	*p* = 0.85
Intracellular fluid (ICF) (in liters)	−0.27	−0.23
	*p* = 0.09	*p* = 0.24
Intracellular fluid (ICF%)	−0.53	−0.04
	*p* < 0.01	*p* = 0.85
Fat mass (FM) (in kg)	−0.24	−0.53
	*p* = 0.14	*p* < 0.01
Fat mass (FM%)	−0.26	−0.48
	*p* = 0.11	*p* = 0.01
Fat free mass (FFM) (in kg)	−0.06	−0.24
	*p* = 0.70	*p* = 0.23
Fat free mass (FFM%)	0.26	0.48
	*p* = 0.11	*p* = 0.01
Albumin (ALB) (in g/dL)	−0.43	−0.18
	*p* < 0.01	*p* = 0.35
Body mass index (BMI) (kg/m^2^)	−0.12	−0.46
	*p* = 0.46	*p* = 0.01
Body weight (in kg)	−0.16	−0.51
	*p* = 0.34	*p* < 0.01
